# Predictive factors of right paraesophageal lymph node metastasis in papillary thyroid carcinoma: Single center experience and meta-analysis

**DOI:** 10.1371/journal.pone.0177956

**Published:** 2017-05-17

**Authors:** Young Min Park, Sang Min Lee, Dong Won Kim, Sung-Chan Shin, Byung-Joo Lee

**Affiliations:** 1 Department of Otorhinolaryngology-Head and Neck Surgery, Korea University College of Medicine, Seoul, Korea; 2 Department of Otorhinolaryngology, Bundang Jesaeng Hospital, Deajin Medical Center, Seongnam, Gyeonggi, Korea; 3 D epartment of Otorhinolaryngology-Head and Neck Surgery, Pusan National University School of Medicine and Biomedical Research Institute, Busan, Korea; University of South Alabama Mitchell Cancer Institute, UNITED STATES

## Abstract

**Background:**

We performed this retrospective study to identify predictors of right paraesophageal lymph node metastasis, and reviewed previous studies related to this topic.

**Methods:**

Between June 2005 and March 2015, 1107 patients were diagnosed with papillary thyroid carcinoma and underwent surgery at Pusan National University Hospital.

**Results:**

Right paraesophageal lymph node metastasis was observed in 171 (15.4%) patients. Multivariate analyses showed that the risk of right paraesophageal metastasis was significantly associated with tumor size, location, a higher number of metastatic central lymph nodes, and lateral lymph node metastasis. In a meta-analysis of the eligible studies, tumor size, number of metastatic central lymph nodes, and lateral lymph node metastasis showed significant relationships with the risk of right paraesophageal metastasis.

**Conclusions:**

In patients with risk factors such as those identified in our study, the possibility of right paraesophageal metastasis should be kept in mind, and careful inspection and dissection are required.

## Introduction

Papillary thyroid carcinoma (PTC) is the most common malignancy originating from the thyroid gland. Although PTC generally shows a favorable prognosis, a subset of PTC showed aggressive features in some patients [1,2]. After initial treatment, the disease relapses in approximately 30% of PTC patients, and reoperation performed to remove the recurrent disease can increase operation-associated morbidity in those patients [3–6]. Lymphatic spread of PTC occurs primarily in the central compartment, including the precricoid, pretracheal, and paratracheal lymph nodes and, in the case of cervical lymph node metastasis, the possibility of loco-regional recurrence increases [3,6,7]. More fibrofatty tissue and lymph nodes are located on the right side of the central compartment because of the different courses along which the recurrent laryngeal nerve (RLN) runs, *i*.*e*., to the left or right sides. In particular, some lymph nodes are present within the space that is surrounded by the right RLN, esophagus, and prevertebral fascia, and are referred to as the right paraesophageal lymph nodes (RPELNs). During central neck dissection (CND), RPELNs may be overlooked because of their deeper anatomical location, and this oversight can lead to regional recurrence.

Cervical lymph node metastasis occurs in 20–90% of PTC patients, and 74% of those recurrent cases experience nodal recurrence in the central compartment [3,8,9]. However, some controversy still exists over the role of prophylactic CND and incomplete lymph node dissection can increase the recurrence, reoperation, and surgical complication rates [3,10–13]. In particular, if the disease relapses in RPELNs located underneath the right RLN, careful dissection is required because of the high likelihood of damage to the RLN. Therefore, to prevent nodal recurrence in this deep-seated location, particular attention and complete dissection are required during the initial surgery for patients with a risk of RPELN metastasis. However, research investigating the risk factors predicting RPELN metastasis is currently insufficient. Therefore, we performed this retrospective study to identify predictors of RPELN metastasis and reviewed previous studies on this topic.

## Materials and methods

Between June 2005 and March 2015, 1107 patients (162 males, 945 females; average age 49 years) were diagnosed with PTC and underwent surgery at Pusan National University Hospital. In all patients, thyroid function tests, thyroid ultrasonography, ultrasonography-guided fine needle aspiration and computed tomography were performed preoperatively. This study was approved the Institutional Review Board of Pusan National University Hospital and informed consent was obtained from all patients.

The ultimate extent of surgery was a total thyroidectomy with bilateral CND in 840 cases, total thyroidectomy with right CND in 265 cases, and right thyroid lobectomy with right CND in 2 cases. Modified radical neck dissection was performed in 216 patients with N1b disease.

The central compartment includes the area between common carotid arteries; it is surrounded by the hyoid bone superiorly, innominate artery inferiorly, and the prevertebral fascia dorsally. The central compartment lymph nodes are divided into the left and right sides according to the midline. During CND procedure, the fibrofatty tissues are dissected off RLN and usually reflected medially, and then dissected off the trachea. The paratracheal nodes may be anterior and posterior to RLN on the right side because of anatomic variation. Additional nodal contents (RPELN) that are deep to the right RLN were removed separately. RPELN were not included as right central lymph nodes and were analyzed separately.

The relationship between the following clinical parameters and RPELN metastasis was analyzed: age, sex, tumor location, tumor size, multiplicity, capsule invasion, number of metastatic central lymph nodes, and number of metastatic lateral lymph nodes. Cases in which at least one lesion was located in the right thyroid lobe were defined as the right lobe lesion group. In cases of multiple lesions, the size and location of the largest lesion was analyzed. Based on the operation and medical records, postoperative RLN injury was investigated.

### Meta-analysis

A systemic literature search was performed in the PubMed, EMBASE, and Cochrane library databases on February 16, 2016. The following terms were used to search for relevant articles: “papillary thyroid carcinoma”, “central lymph node”, and “paraesophageal lymph node”. No other conditions were applied during the search. The retrieved articles were evaluated manually, and the references within these articles were also searched. Duplicate articles were excluded based on title, first author’s name, and year of publication.

Articles that met the following criteria were included in this study: (1) randomized or nonrandomized controlled trials/prospective or retrospective studies; (2) studies involving patients diagnosed with PTC who received thyroidectomy and lymph node dissection; (3) studies including data regarding central lymph nodes and RPELNs; (4) studies in which lymph node metastasis was evaluated based on pathologic examination. Two reviewers analyzed the titles and abstracts of the retrieved articles during the initial screening. If there were discrepancies between the two independent reviewers, they were resolved through discussion or by referral to a third reviewer.

The following data were extracted from the selected articles: first author’s name, year of publication, country, study period, study design, patients’ clinical information, type of surgery performed, patient number, number of patients with/without central lymph node metastasis or RPELN metastasis, odd ratios (ORs), and 95% confidence intervals (CIs).

### Statistical analysis

The chi-square or Fisher’s exact test was used to evaluate differences in categorical variables between two independent groups. An independent two-sample *t-*test was used to assess differences in continuous variables between two independent groups. Risk factors for RPELNs were identified using logistic regression models. Statistical analyses were performed using SPSS 18.0 for Windows (SPSS, Chicago, IL). For the meta-analysis of previous studies, both the χ^2^-based Q statistic and the I2 statistic were used to assess the heterogeneity of the studies. In the absence of heterogeneity (*P*>0.10, I2<25%), pooled estimates of the odds ratios (OR) with their 95% confidence intervals (CI) were calculated via the fixed effects model. In the presence of heterogeneity (P<0.10, I2>25%), the random effects model (DerSimonian and Laird method) was used to pool the primary study estimates. The statistical analyses for the meta-analysis were performed using RevMan 5.3. A *p*-value <0.05 was considered to indicate statistical significance.

## Results

Of all 1107 patients, 746 (67.4%) patients had the lesion on right lobe. The mean size of the primary tumor on the right lobe was 10.1mm (range, 1–54mm). And 361 (32.6%) patients had the lesion on left lobe and the mean size of the primary tumor was 10.2mm (range, 1–125mm). During the study period, prophylactic central neck dissection was performed in 800 (72.3%) cases and therapeutic central neck dissection was performed 307 (27.7%) cases. Central lymph node metastasis was found in 689 (62.6%) of the 1107 patients. Right central lymph node metastasis was observed in 543 (49.1%) patients, left central lymph node metastasis was found in 448 (40.5%) patients. Bilateral central lymph node metastasis was found in 216 (19.5%) patients and 19 (1.7%) patients had only contralateral central lymph node metastasis without ipsilateral metastasis. RPELN metastasis was found in 171 (15.4%) patients. Of the 171 patients with RPELN metastasis, 151 (88.3%) also had other central lymph node metastasis. Of the 216 patients with lateral lymph node metastasis, 72 (33.3%) had RPELN metastasis, 65 (90.3%) of whom also had right central lymph node metastasis.

Univariate analyses were performed between the 171 patients with RPELN metastasis and 936 patients without RPELN metastasis. Age and multiplicity were not significantly associated with RPELN metastasis. The rate of RPELN metastasis was significantly higher in males than in females (22.8% vs. 14.1%, *p* = 0.005). The rates of RPELN metastasis were 9.8%, 21.4%, and 35.6% in tumors <1 cm, 1–2 cm, and >2 cm, respectively (*p*<0.0001, χ^2^ test). Lesions located in the right lobe had a higher rate of RPELN metastasis than lesions in non-right lobe (77.8% vs. 22.2%, *p* = 0.002, χ^2^ test). The rate of RPELN metastasis was significantly higher in cases with capsular invasion than in cases without capsular invasion (64.9% vs. 35.1%, *p =* 0.005, χ^2^ test). Patients with 0, 1–2, or ≥3 metastatic central lymph nodes had RPELN metastasis rates of 4.8%, 12.7%, and 30.4%, respectively (*p*<0.0001, χ^2^ test). Patients with 0, 1–2, or ≥3 metastatic lateral lymph nodes had RPELN metastasis rates of 11.1%, 28.8%, and 36.4%, respectively (*p* = <0.0001, χ^2^ test) (Table 1).

**Table 1:** Clinicopathological characteristics of patients with or without RPELN metastasis.

	RPELN metastasis (–) n = 936, n (%)	RPELN metastasis (+) n = 171, n (%)	*p*-value
Sex			
Male	125 (13.4)	37 (21.6)	
Female	811 (86.6)	134 (78.4)	
Age			
Mean±SD	48.9±12.1	49.0±13.8	0.879
<45 years	350 (37.4)	68 (39.8)	0.556
≥45 years	586 (60.6)	103 (60.2)	
Diameter			<0.0001
≤1 cm	629 (67.2)	69 (40.4)	
1–2 cm	242 (25.9)	66 (38.6)	
>2 cm	65 (6.9)	36 (21.0)	
Multiplicity			0.858
Yes	186 (19.9)	35 (20.5)	
No	750 (80.1)	136 (79.5)	
Tumor location			0.002
Right lobe	613 (65.6)	133 (77.8)	
Non-right lobe	323 (34.5)	38 (22.2)	
Capsule invasion			0.005
Yes	499 (53.3)	111 (64.9)	
No	437 (46.7)	60 (35.1)	
No. of meta-CLN			<0.0001
0	398 (42.5)	20 (11.7)	
1–2	289 (30.9)	42 (24.6)	
≥3	249 (26.6)	109 (63.7)	
No. of meta-LLN			<0.0001
0	798 (85.3)	100 (58.5)	
1–2	47 (5.0)	19 (11.1)	
≥3	91 (9.7)	52 (30.4)	

RPELNs, right paraesophageal lymph nodes; SD, standard deviation; meta-CLN, metastatic central compartment lymph nodes; meta-LLN, metastatic lateral compartment lymph nodes.

Multivariate analyses showed that the rate of RPELN metastasis significantly increased with tumor size, location, a higher number of metastatic central lymph nodes, and lateral lymph node metastasis (Table 2).

**Table 2:** Multivariate analysis of the risk factors for RPELN metastasis.

	OR	95% CI	*p*-value
Tumor size	1.693	1.312–2.186	<0.0001
Location	1.949	1.291–2.941	0.001
No. of meta-CLN			
1–2	0.158	0.092–0.271	<0.0001
≥3	0.402	0.265–0.611	<0.0001
Meta-LLN	1.470	1.174–1.841	0.001

RPELN, right paraesophageal lymph node; OR, odds ratio; CI, confidence interval; meta-CLN, metastatic central compartment lymph nodes; meta-LLN, metastatic lateral compartment lymph nodes.

RLN injury was observed in 9 of the 1107 patients. Eight of the nine patients recovered within 1–6 months postoperatively. One patient received an intracordal injection of hyaluronic acid for permanent vocal cord palsy after 6 months postoperatively.

**Fig 1:**
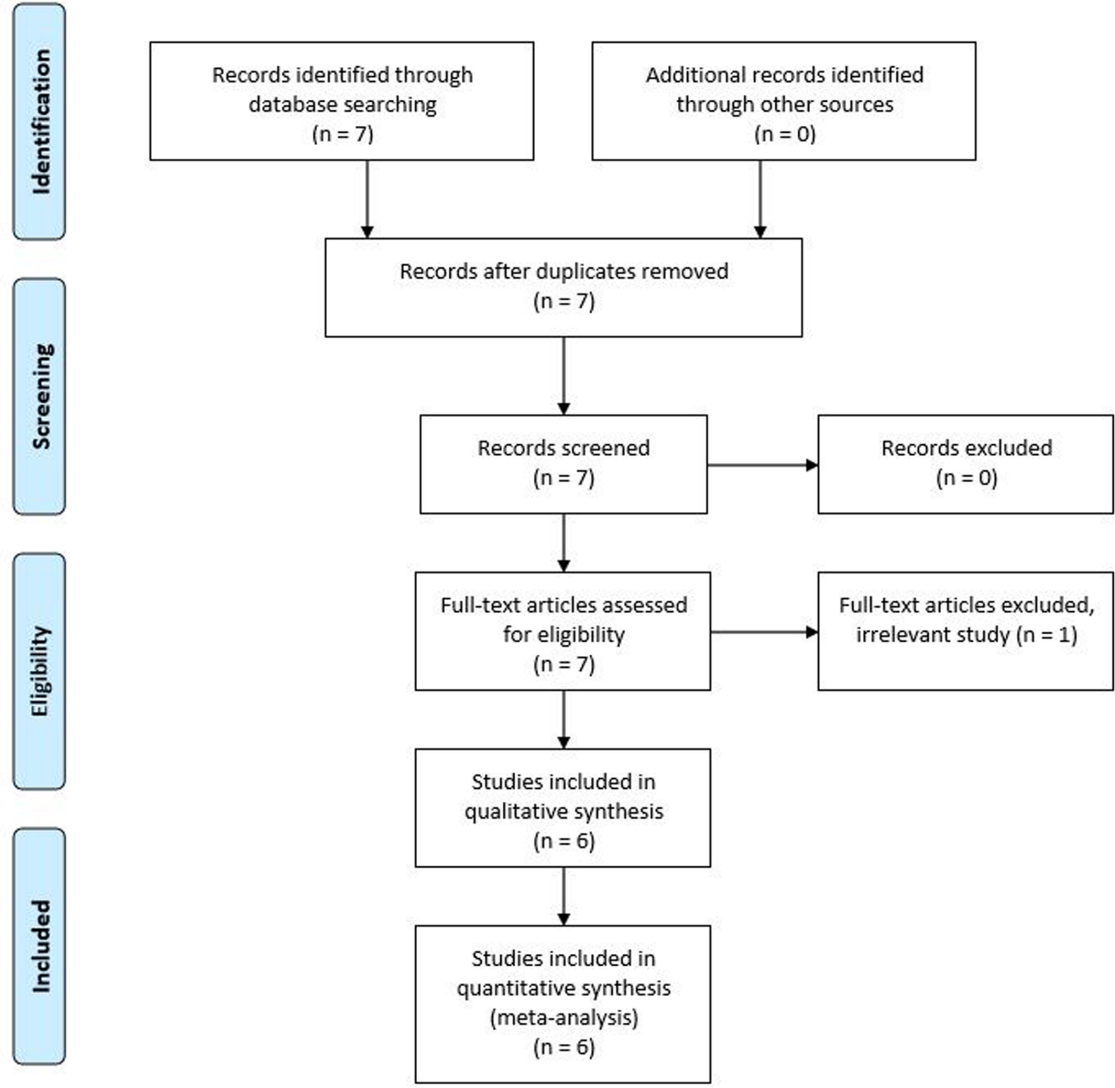
Flow chart of study selection.

The six eligible studies in this meta-analysis were found through database searches (Fig 1 and Table 3). These articles were published from 2009 to 2016, and all of the studies were designed retrospectively. Three studies were conducted in Korea, two in China, and one in Japan. A total of 2308 patients were included, and the number of patients in each study ranged from 123 to 922. The mean or median age of the patients ranged from 44.3 to 52.8 years, and the female to male ratio ranged from 3.4:1 to 5.7:1. The prevalence of RPELN metastasis ranged from 5.8% to 26.7%. Regarding the surgical procedure, CND including RPELN dissection was performed in all of the studies, and the most common surgical types performed were: total thyroidectomy in two studies, hemithyroidectomy or total thyroidectomy in two studies, and hemithyroidectomy, subtotal thyroidectomy, or total thyroidectomy in two studies. In four studies, modified radical neck dissection was performed only in PTC patients with lateral neck node metastasis. On the other hand, in another study, prophylactic modified radical neck dissection was performed in selected cases.

**Table 3:** Meta-analysis of six studies of RPELN metastasis in PTC.

Author	Year	Country	Study period	Study design	Patients n	Prevalence of RPELN metastasis %
Zhang *et al*.	2016	China	Sep 2010–Aug 2013	Retrospective	246	13.4
Pinyi *et al*.	2014	China	Jan 2010–Jun 2012	Retrospective	405	26.7
Ito *et al*.	2013	Japan	Jan 2004–Dec 2009	Retrospective	922	13.8
Kim and Park	2012	Korea	Apr 2008–Jan 2010	Retrospective	243	5.8
Bae *et al*.	2012	Korea	Aug 2008–Jan 2010	Retrospective	369	12.2
Lee *et al*.	2009	Korea	Mar 2007–Feb 2008	Retrospective	123	11.4

RPELN, right paraesophageal lymph node.

The proportion of males was significantly higher in the groups with RPELN metastasis than in groups without RPELN metastasis, with a significant association found between male sex and RPELN metastasis (OR 1.79, 95% CI 1.36–2.36, *p<*0.001, fixed effects model). No significant association was found between age and RPELN metastasis (OR –0.85, 95% CI –3.43–1.72, *p =* 0.52, fixed effects model). Tumor size (>1 cm) was found to be significantly associated with RPELN metastasis (OR 4.20, 95% CI 3.0–5.9, *p<*0.001, fixed effects model). Capsular invasion was significantly associated with RPELN metastasis (OR 2.64, 95% CI 1.11–6.30, *p =* 0.03, random effects model). The number of metastatic central lymph nodes was significantly associated with RPELN metastasis in the analysis of four studies (OR 5.33, 95% CI 2.40–8.26, *p<*0.00001, random effects model). Lateral lymph node metastasis was significantly associated with RPELN metastasis in the analysis of four studies including 1143 patients (OR 6.61, 95% CI 4.48–9.75, *p<*0.0001, fixed effects model).

## Discussion

PTC commonly metastasizes to the cervical lymph nodes, and nodal metastasis occurs primarily in the central compartment lymph nodes, including the prelaryngeal, pretracheal, and paratracheal lymph nodes. However, given the different courses along which the RLN runs, to the left or right side, RPELNs are located in the space that is surrounded by the right RLN, esophagus, and prevertebral fascia. RPELNs located in this deep-seated anatomical area, which are difficult to access, could be overlooked during CND. Incomplete dissection of these nodes can be a cause of disease recurrence, and reoperation may increase the patients’ morbidity and postoperative complications. However, the sensitivities of preoperative imaging studies, including ultrasonography and computed tomography, are not sufficient to detect lymph node metastasis of the central compartment [14]. Thus, risk factors that predict RPELN metastasis should be identified, as they may be useful for evaluating the nodal status of PTC patients before surgery and for determining whether lymph node dissection including RPELNs is necessary.

In this study, the rate of RPELN metastasis was 15.4% among the 1107 PTC patients. Tumor size, location, higher number of metastatic central lymph nodes, and lateral lymph node metastasis were significantly associated with RPELN metastasis. Although age is an important prognostic factor for PTC, and cervical lymph node metastasis commonly occurs in patients less than 45 years of age, age was not significantly associated with RPELN metastasis in our study [15]. Although previous studies reported that tumor distribution, such as bilaterality and multifocality was related to cervical lymph node metastasis, our study did not find a significant correlation between tumor multiplicity and RPELN metastasis [15,16]. However, right lobe lesion was significantly associated with a risk of RPELN metastasis. The metastatic nodal status, including extracapsular spread, number of metastatic central lymph nodes, and lateral lymph node metastasis, is a known prognostic factor of PTC [17,18]. Unfortunately, extracapsular spread and metastatic nodal size were not analyzed because of insufficient medical records in this study. However, a higher number of metastatic central lymph nodes had a significant relationship with RPELN metastasis, and patients with lateral lymph node metastasis had a higher rate of RPELN metastasis than patients without metastasis.

The rate of RPELN metastasis in PTC patients ranged from 5.8% to 26.7% in previous studies [19–24]. As mentioned above, RPELN metastasis of PTC is not uncommon, and it has the potential to cause disease recurrence during the follow-up period. Of patients with recurrent or persistent PTC, 57% exhibited metastatic lymph nodes in the central compartment [25]. In addition, Lei et al. reported a higher rate of RPELN metastasis in patients with relapsed disease than in patients with newly diagnosed PTC [19]. The reason is thought to be that RPELNs are not removed completely during the initial surgery due to their deep location underneath the RLN, which makes them difficult to access. In patients with risk factors such as those identified in our study, the possibility of RPELN metastasis should be kept in mind, and careful inspection and dissection are required during surgery.

We reviewed and analyzed six studies related to this topic. Based on the results of a meta-analysis of the eligible studies, tumor size, number of metastatic central lymph nodes, and lateral lymph node metastasis consistently showed significant relationships with the risk of RPELNs. In particular, patients with >3 metastatic central lymph nodes had a higher rate of RPELN metastasis. Accordingly, in cases with several suspicious lymph nodes in the central or lateral compartment, the extent of central lymph node dissection must include RPELN. Although it is not easy to detect all metastatic lymph nodes preoperatively with current imaging studies, RPELN should be removed in patients suspected of having metastatic lymph node diseases [19].

## Conclusion

In patients with risk factors such as those identified in our study, the possibility of right paraesophageal metastasis should be kept in mind, and careful inspection and dissection are required during surgery.

## Supporting information

S1 Text PRISMA checklist. (PDF)
